# Supplementary material for: Loss of Farnesoid X receptor (FXR) accelerates dysregulated glucose and renal injury in db/db mice
Source: PeerJ. 2023 Sep 29;11:e16155. doi: 10.7717/peerj.16155 (PMC10544308; doi:10.7717/peerj.16155)
Supplement: Supplemental Information 2 [file peerj-11-16155-s002.docx]

**Supplementary Figures**

**Fig S1**


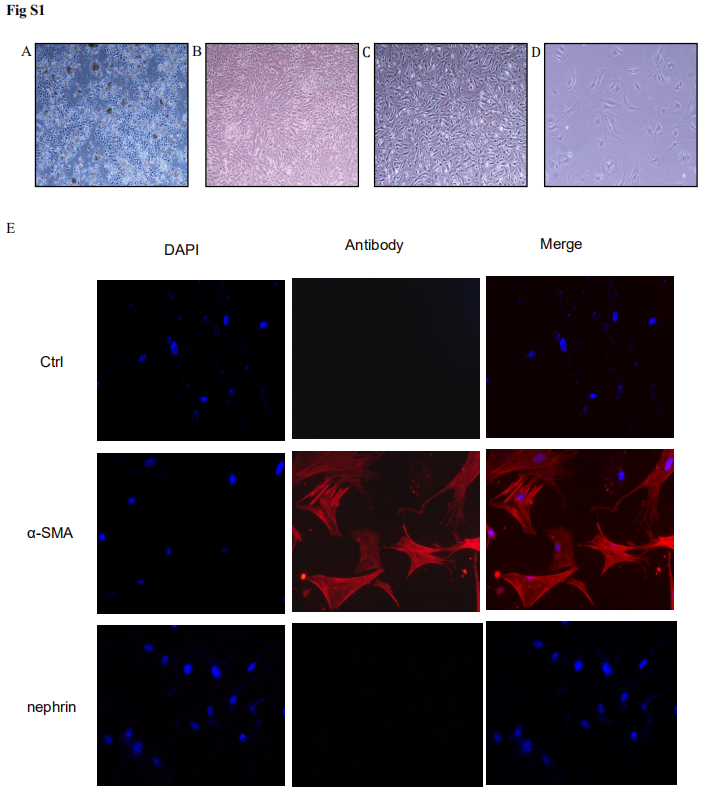


Fig S1. Mouse primary mesangial cells culture and identification. (A) On the first three days of culture, the cells showing glomeruli have been adherent; (B) On the seventh day of culture, P1 generation is about to pass, we can see a large number epithelium and endothelial cells (C) On the 21st day of culture, a large amount of epithelium and endothelial cells have died, P-MC has climbed out and was to be purified; (D) On the 25th day of culture, P-MC has purified; (E) P-MC Immunofluorescence Assay Map: P-MC was shown for IgG (first line), α-SMA (second line) and nephrin (third line). The blue DAPI represents the nucleus (first column), the red staining represents cells other than the nucleus (second column) and the third column represents the merge map. P-MC can dye the specific antibody α-SMA, but not the specific antibody nephrin of podocytes.

**Fig S2**


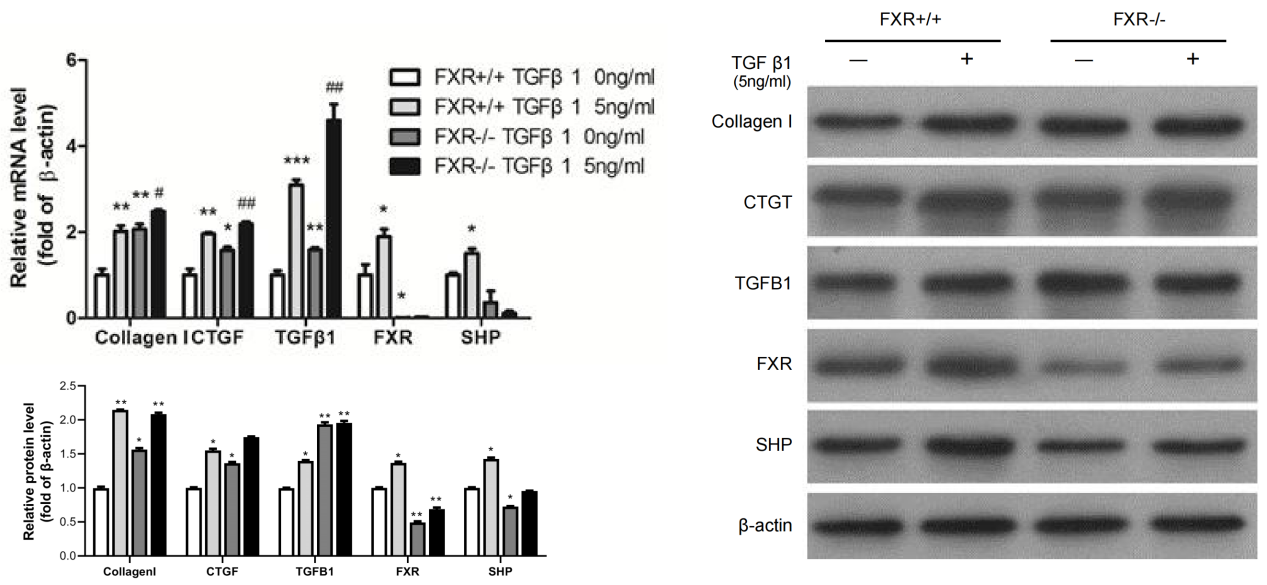


Fig S2. After treatment TGFβ1 (5 ng/ml) for 24h on P-MC, the cells were from FXR^+/+^ and FXR^-/-^ mice, the mRNA levels of Collagen Ⅰ, CTGF, TGFβ1, FXR, and SHP were detected by qRT-PCR (n=3) and the protein levels were examined by western blot (n=3). *p<0.05, **p<0.01, ***p<0.001 vs. FXR^+/+^ TGFβ1 0 ng/ml. #p<0.05, ##p<0.01vs. FXR^-/-^ TGFβ1 0 ng/ml.

**Fig S3**


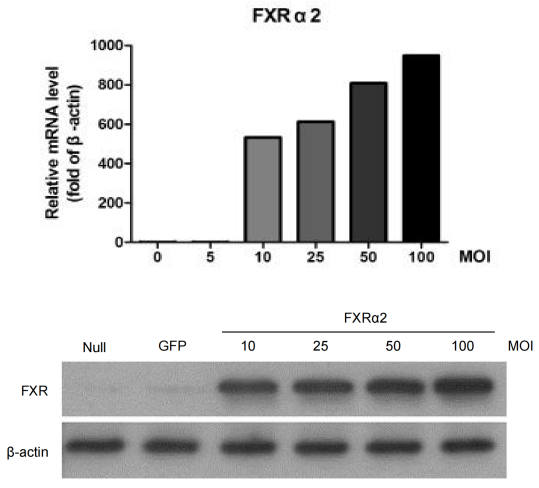


Fig S3. The verification of FXR adenovirus α2. After treatment adv-FXRα2 for 24h on P-MC, the mRNA levels of FXR were detected by qRT-PCR and the protein level of FXR was examined by Western blot(n=1).

**Fig S4**


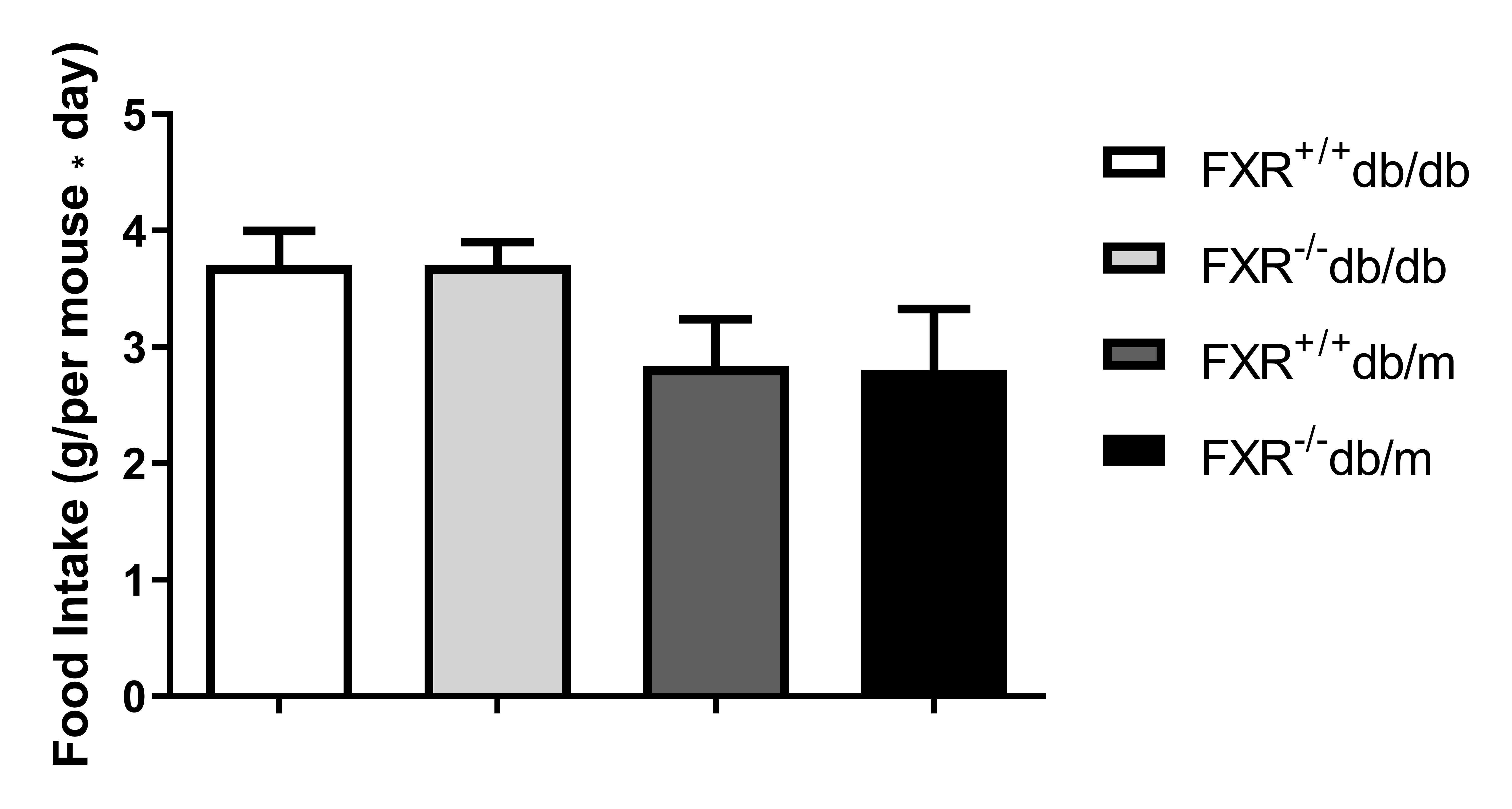


Fig S4. Daily food intake each mouse was measured in four type mice (n=3).
